# Spider Monkeys Rule the Roost: Ateline Sleeping Sites Influence Rainforest Heterogeneity

**DOI:** 10.3390/ani9121052

**Published:** 2019-12-01

**Authors:** Andrew Whitworth, Lawrence Whittaker, Ruthmery Pillco Huarcaya, Eleanor Flatt, Marvin Lopez Morales, Danielle Connor, Marina Garrido Priego, Adrian Forsyth, Chris Beirne

**Affiliations:** 1 Osa Conservation, Conservation Science Team, Washington, DC, 20005, USA; LawrenceWhittaker@hotmail.co.uk (L.W.); ruthp@osaconservation.org (R.P.H.); eleanorflatt@osaconservation.org (E.F.); marvinlopez@osaconservation.org (M.L.M.); marinagarrido@osaconservation.org (M.G.P.); adrianforsyth@gmail.com (A.F.); 2 Institute of Biodiversity, Animal Health and Comparative Medicine, College of Medical, Veterinary and Life Sciences, University of Glasgow, Glasgow, G12 8QQ, UK; 3 Division of Biology, Imperial College London, Silwood Park Campus, Ascot, Berkshire SL5 7PY, UK; 4 Centre for Ecology & Conservation, School of Bio sciences, University of Exeter, Penryn Campus, Cornwall, TR10 9FE, UK; daniconnor1995@gmail.com (D.C.); c.w.beirne@gmail.com (C.B.)

**Keywords:** rainforest, wildlife, camera traps, Ateline, primates, biodiversity, seed dispersal, seed predation, trophic interactions

## Abstract

**Simple Summary:**

Spider monkeys are important dispersers of many hardwood trees that contribute greatly to the carbon sequestration of tropical forests. One way in which Spider monkeys influence tropical ecosystem structure and function is through the creation of visible terrestrial latrines beneath their “sleeping sites”—trees in which they frequently return to sleep. Spider monkey latrines are thought to create high quality resource patches for rainforest plants and other wildlife to exploit. We investigate this using camera traps placed in both the canopy and on the rainforest floor to determine which rainforest wildlife are attracted to the latrines beneath the sleeping sites of spider monkeys. We also assess the tree species and dung beetles found within the latrines compared with other areas of the forest. Our evidence suggests that spider monkey roosting sites are a hub of activity for other rainforest wildlife, and act as germinating beds for many rainforest trees. If rainforests were to lose spider monkeys, from intensive hunting for example, many other rainforest wildlife species would be affected, and forests would therefore be made up of different tree communities than landscapes where spider monkeys exist.

**Abstract:**

The sleeping site behavior of Ateline primates has been of interest since the 1980s, yet limited focus has been given to their influence upon other rainforest species. Here, we use a combination of arboreal and terrestrial camera traps, and dung beetle pitfall traps, to characterize spider monkey sleeping site use and quantify the impact of their associated latrines on terrestrial vertebrate and dung beetle activity. We also characterize the physical characteristics of the sleeping sites and the floristic and soil composition of latrines beneath them. Spider monkey activity at sleeping sites peaked at dawn and dusk and group composition varied by sex of the adults detected. The habitat-use of terrestrial fauna (vertebrates and dung beetles) differed between latrine sites and non-latrine controls, underpinned by species-specific changes in the relative abundance of several seed-dispersing species (such as paca and great curassow). Seedling density was higher in latrines than in non-latrine controls. Although most soil properties were similar between latrines and controls, potassium and manganese concentrations were different. These results suggest that spider monkey sleeping site fidelity leads to a hotspot of ecological activity in latrines and downstream impacts on rainforest floristic composition and diversity.

## 1. Introduction

The Central American or Geoffroy’s spider monkey (*Ateles geofroyii*) is an endangered Ateline primate and a major disperser of large-seeded hardwood trees, which contribute substantially to the biomass and carbon storage value of tropical forests [1,2]. Consequently, the loss of spider monkeys from ecosystems can cause a cascading breakdown in ecological interactions [3], resulting in changes in floristic composition and reductions in carbon sequestration [2,4]. The principal way in which spider monkeys are thought to influence tropical forest structure is through the dispersal of seeds via defecation as they forage through the forest (e.g., Link and Di Fiore [5]). However, spider monkeys can also influence forest structure in more cryptic, less well-studied ways. For example, spider monkeys use specific trees for sleeping, which promotes aggregated deposition of large volumes of seeds via feces from a variety of different tree species [6].

The sleeping site behavior of primates has been of interest to tropical ecologists since the 1950s [7], and that of Ateline primates since the 1980s [8]. The sleeping patterns of Atelines have been shown to relate directly to the distribution and availability of food resources within surrounding forest habitat [9]. When food resources are scarce, spider monkeys show low sleeping site fidelity (the frequency of use of sleeping sites), typically using a given location for a single night; likely to avoid unnecessary use of energy in excessive travel and to remain close to a food resource that could otherwise be exploited by competitors [9]. However, when food is abundant, spider monkeys show stronger sleeping site fidelity, regularly traveling to and aggregating in large trees at dusk. Fidelity of sleeping sites appears sex-specific, where adult males are less likely to be found at these regular sites than females and juveniles, and that all-male sub-groups use regular sites less frequently [10]. It is beneath the regularly frequented sites that large distinct latrines can be observed on the forest floor. Latrines are created as group members high in the canopy regularly defecate on the ground below, typically in the early mornings, leaving behind a hub of seeds, nutrients, and germinating seedlings [11].

Although movement behavior of spider monkeys has received substantial research attention [12], relatively limited focus has been given to the characterization of sleeping sites [11] and how they influence local floristic composition [13] via seedling survival and dispersal mechanisms [5,9]. It has been shown that sleeping site fidelity is positively associated with seed abundance, plant species diversity, and species turnover, yet negatively correlated to seed community evenness [6]. Sleeping site fidelity has previously been defined and assessed either by the labor-intensive following of monkeys, or based on feces presence rates at monthly check intervals when emptying seed traps located below sleeping sites [6]. However, such methods are unlikely to accurately reflect the intensity of sleeping site use, partly due to that fact that ground-based observations are limited in their capacity to observe what is occurring in the upper canopy, especially at night-time and because continuous monitoring of multiple latrines is logistically challenging. One potential method to address the limitations of previous approaches is the use of arboreal camera trapping [14,15,16]. Camera traps placed in the canopy provide direct and relatively unobstructed records of activity in the canopy, provide the benefit of operating over long time frames (over months), record activity both night and day, and have been proven effective in behavioral and biodiversity studies within rainforest canopies [17,18,19].

While we are aware of the importance of latrines in shaping floristic composition of the forest floor [6,20,21], it has also been suggested that spider monkey latrines present a large and diverse food resource that might be attractive to seed predators and secondary dispersers [13]. Given that latrines represent high-quality resource patches with greater seed and fruit densities, we would expect greater numbers of seed dispersers, seed predators, and frugivores to aggregate beneath spider monkey sleeping sites [22]. We are not aware of any research to date which has directly studied the effect of latrines on other rainforest wildlife visitation rates. This is another aspect of spider monkey latrine ecology than can be investigated using remote camera traps to target the visitation of vertebrate seed predators and dispersers. In addition to the potential link between spider monkey latrines and terrestrial vertebrate activity, we would expect that dung beetles are also strongly associated with rainforest latrine sites created by spider monkeys [23,24]. Dung beetles are secondary dispersers of rainforest seeds, responsible for both moving them away from parent trees to escape density-dependent mortality [25,26,27], and in burying seeds, which can help to avoid predation by rodents and increase germination success [28,29].

Here, we carry out the first camera trap study of spider monkey sleeping sites within the lowland wet tropical forests of the Osa Peninsula, on the Pacific coast of Costa Rica. Specifically, we: (1) Use arboreal camera traps to quantify the intensity of use of sleeping sites by spider monkeys; (2) use terrestrial camera traps to determine the effect of sleeping site latrines on the presence on terrestrial vertebrates; (3) use pitfall traps to determine the effect of sleeping sites on dung beetles; and (4) use floristic and soil nutrient assessments to assess the physical characteristics of the forest surrounding sleeping sites. Our findings highlight cascading ecological impacts of latrine sites on terrestrial vertebrate and invertebrate communities and suggest ways in which the presence of latrines might generate heterogeneity in forest composition and soil quality.

## 2. Materials and Methods

### 2.1. Study Site

The Osa Peninsula in southwest Costa Rica is home to the largest remaining tract of Pacific lowland wet forest in Mesoamerica [30] and hosts four protected areas—Corcovado and Piedras Blancas National Parks, the Terraba del Sierpe Wetland, and the Reserva Forestal Golfo Dulce. Corcovado National Park is home to one of the largest populations of the endangered Central American spider monkey (*Ateles geoffroyi*); with a population density estimate calculated from transect surveys in 2002–2003 of 68.45 (±26.25) individuals/km^2^ [31]. The park encompasses 424 km² with a matrix of primary, secondary, and coastal forest [32,33,34]. Since the protected area was established in 1975, pressure from illegal hunting and logging has been largely eradicated in the park.

**Figure 1:**
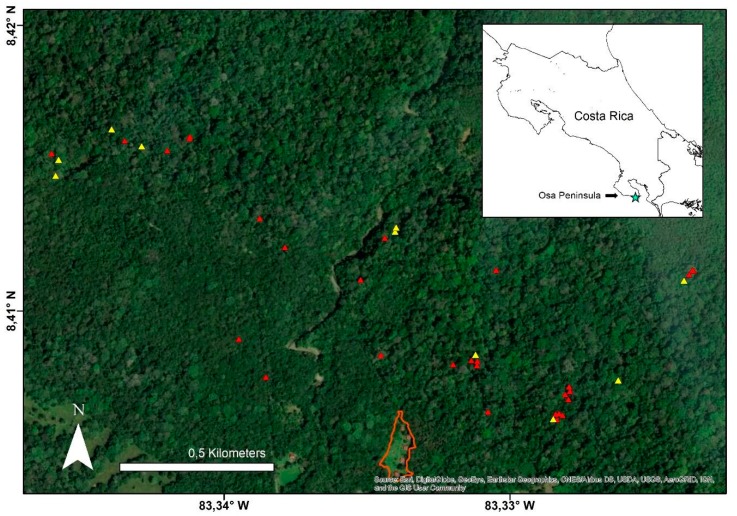
Map of the study area. The Osa Biological Station is outlined in orange, and the 39 identified sleeping sites as part of the current study are represented by triangles; the 10 focal trees for investigation are represented by yellow triangles, all others by red. The inlay shows the location of the Osa Peninsula in the southwest pacific of Costa Rica, and the study location on the peninsula represented by the blue star.

Our study site was situated around the Osa Biological Station (formerly known as Piro Biological Station; 8.40388 N, 83.33661 W; see Figure 1), located in a biological corridor within the Reserva Forestal Golfo Dulce. The reserve directly surrounding the station is comprised of 1330 ha of privately protected land with a variety of habitat types, including old-growth primary forest, naturally regenerating secondary-growth forest, secondary plantation forest (cattle pastures that were converted to monoculture plantations ~30 years ago, enriched by planting 80,000 trees of 50 native species), and an active agricultural matrix (for a detailed description, see Whitworth et al. [35]). Hunting has been eradicated for at least the last 17 years, when the land was acquired by N.G.O Osa Conservation.

### 2.2. Sleeping Site Identification

Sleeping sites were located by following subgroups of *Ateles geofroyii* during transects performed throughout the early wet season (June to September) of 2017. Individuals of these subgroups typically began traveling to sleeping sites after 17:00 and settled around 18:00. Before sunrise the following morning, we returned to the sleeping site in order to minimize disturbance and prevent monkeys fleeing the tree before defecation. Spider monkeys typically started their activity at 04:50 and, on most occasions, had left the tree by 05:15, by which time they had defecated. Once the individuals had left, the area was searched to locate latrines. Latrines were identified in the field by three key features: (1) The presence of spider monkey feces; (2) a higher than normal density of young saplings; and (3) clear evidence of excreted seeds in the leaf litter immediately beneath the sleeping site. Latrines were difficult to identify after a night of rain, as feces were typically washed away. At each sleeping, site we measured the following variables: Canopy height (m), canopy cover (%), diameter at breast height (cm), tree species (of the focal tree), and we mapped out the latrines using a GPS. Of the 39 sleeping sites identified, 10 of the most active (based on perceived continued sleeping site activity throughout the early dry season) were selected for a multi-strata assessment using three camera traps at each site. The camera traps were set up at each site between February and June 2018.

### 2.3. Characterizing Sleeping Site Usage with Arboreal Camera Traps

In order to monitor spider monkey sleeping site use and behavior, one camera trap was placed in the canopy, either facing along a horizontal branch of the sleeping site, or when trees were unable to be climbed for safety reasons, facing at the whole sleeping site from a nearby suitable tree. Ideally, we would have more cameras in a tree to increase detection likelihood, just as every ground-based survey would want more observers on the ground to spot, count, and sex monkeys; however, it is not always logistically possible (cost and time being the principal issues). Having a single camera at a survey site is a common limitation in the camera trap literature generally, but having at least one camera provided us with a long-term constant monitoring tool within the sleeping site, and we tried to ensure it was directed to where we had observed spider monkeys using the tree, based both on direct observations and upon the location of the latrine on the ground below. All cameras were programmed to take 13 s videos with a 30 s resting period to maximize battery life [14,36,37]. To gather information on spider monkey sleeping site group structure and behavior, the number of individuals, sex of individuals (male, female, or unknown), and age class (adult or juvenile) were determined from each camera trap video where possible. We did not try to determine the sex of the juveniles as it was not possible from video footage. In terms of sexing rates, we transparently report our sample sizes and the number of individuals it was possible to sex, and those that it was not. Given the difficulty in observing group compositions from the ground this is a clear advance in our current understanding.

### 2.4. Characterizing Latrine Use by Terrestrial Vertebrates

To gather information on terrestrial seed dispersers and predators, mammal and bird species were identified from the terrestrial camera footage. Camera traps were deployed in a paired design, with the first camera placed at ~30 cm height from the ground directed at the latrine, and the second camera placed 50 m away at the same height, at a random forest point (non-latrine control site). The detection zone of camera traps easily covered the average core area of latrines. Although all methods suffer from imperfect detection, particularly within tropical forests, one of the strengths of this survey is that we used a standardized method of surveying to examine habitat use within latrines in comparison with paired non-latrine controls. Independent records were defined by 30-min intervals, with any of the same species within 30 min being regarded as non-independent (as in Pillco et al. [38]). We then calculated the independent visitation rate across camera sites (independent visits per 30 trap days). Species richness and visitation rates in both latrine and non-latrine control sites were also estimated.

### 2.5. Characterizing Latrine Use by Dung Beetles

To determine the effects of latrine presence on dung beetle assemblages, pitfall sampling was carried out from August to December 2018. This was done after the camera trapping survey was completed to minimize anthropogenic disturbance at the site while cameras were active. At each latrine and non-latrine control site, pitfall traps were set baited with 25 g of human dung [39,40]. We chose to use human dung as prior research in the Neotropics has shown that the attractiveness to a bait to dung beetles was greatest for human dung and there appeared to be no specificity between human and primate dung [41]. In addition, human dung is used to standardize collecting methods because it is readily available at any study site in the world and is among the most attractive types of dung to most species of dung beetles [39]. While using *Ateles* dung might have been the best-case scenario, finding a sufficient quantity of fresh primate dung was logistically impossible without access to a large captive population. However, as with the terrestrial cameras, we used a standardized method of surveying to examine habitat use within latrines in comparison to paired non-latrine controls. The only thing that differed between the latrine site and the non-latrine sites was the presence of the latrine. Everything else was standardized (as much as is possible within a complex tropical ecosystem), so we were therefore confident that any differences observed were being driven by the presence of the Ateline sleeping site latrines and not the attractant. Traps were set for three rounds of 24-h trapping. At each location, the latrine and control traps were surveyed simultaneously for a direct comparison over the same time period. Dung beetles were trapped into alcohol for processing and identification. The abundance, species richness, and biomass of trap sites were determined. Biomass was calculated by multiplying the abundance of each respective species by the known average dry biomass calculated for each species and then aggregating to display the overall trap biomass.

### 2.6. Characterizing the Effects of Latrines on Seedling Density and Soil Quality

To quantify the effect of spider monkeys on the surrounding tree seedling density, four 2 m^2^ quadrats were surveyed at each sleeping site. The quadrats were placed within the latrine, beneath the focal tree outside of the latrine, and 4 m from the latrine outside the crown of the tree. The number of tree seedlings was counted in each quadrat.

Soil samples were taken from latrine and the paired non-latrine control sites using a soil extracting device from five points at a depth of 10–15 cm. Before extracting the soil, the top layer of leaf litter was removed. The extracting device was cleaned between sampling points to ensure no cross-contamination. The soil from the five points was extracted into a bucket, mixed, and 500 g was placed in a zip-lock bag and transported to the University of Costa Rica (UCR) laboratory for analysis within 72 h. At the UCR laboratory, the samples were analyzed for micronutrient levels, including: Calcium (Ca), magnesium (Mg), potassium (K) in cmol/L; copper (Cu), iron (Fe), manganese (Mn), and zinc (Zn) in Mg/L. In addition, the percentage of macronutrients carbon (C) and nitrogen (N), and the pH of each sample were determined. We were especially interested in the levels of potassium, phosphorus, and nitrogen, as they are known plant-limiting nutrients from tropical forests [42].

### 2.7. Data Analysis

All data analysis was conducted in the R statistical environment [43]. To characterize diel patterns of sleeping site usage, we used the “activity” package [44] on the raw detection data.

To examine the diel variation in group size, we fitted a loess smoother (span 0.55, degree = 2) to the group size per detection data. To compare the community compositions between latrine sites and non-latrine controls, we used non-metric multidimensional based on Bray–Curtis similarities within the “vegan” package [45]. To determine if the community composition differed, we used the PERMANOVA dissimilarity test, then determined key species driving the proposed differences using the “SIMPER” package [46]. We examined whether latrines influenced general patterns in visitation or capture rates using mixed-effects models, with study site as a random intercept term [47]. For the camera trap data, the response terms were overall and taxon-specific (mammal, bird, reptiles) capture rates (number of independent detections per 30-day period), and for the pitfall trapping data, the response terms were dung beetle species richness, abundance, and biomass. We also confirmed the direction and magnitude of the response of species and soil parameters identified as driving shifts in community composition (identified by “SIMPER”) using linear mixed-effects models with site as a random intercept term. In all cases, we compared the significance of the latrine fixed effect with a nested “null model” without that term using likelihood ratio tests. The relationship between seedling species richness and spider monkey latrine use frequency was determined using a linear model, with latrine use as the explanatory variable and observed seedling richness as the response term.

## 3. Results

Overall, we located 39 sleeping sites and 28 latrines. The 39 sleeping sites belonged to 21 different tree species, 18 genera, and 12 families. In old-growth forest, spider monkeys slept in 25 trees belonging to 13 species and 10 families. In secondary forest, the 14 trees identified were comprised of 11 species and 6 families (see Supplementary Table S1 for details of all tree species and the 10 focal selected trees). The most common species, representing 35% of the 39 sleeping sites identified were *Brosimum utile* (*n* = 3), *Otoba novogranatensis* (*n* = 3), and *Tapirira guianensis* (*n* = 3). The average diameter at breast height of sleeping sites was 59.5 cm (min = 20.7 cm; max = 116 cm). Twenty-eight of the sleeping sites had clear evidence of latrines beneath them. Latrines had a mean area of 5.7 ± 1.8 m^2^. One latrine shared by several sleeping sites had an area of 56.1 m^2^. The 10 sub-selected sleeping sites for further camera and trapping surveys consisted of eight different species.

### 3.1. Use of Sleeping Sites

**Figure 2:**
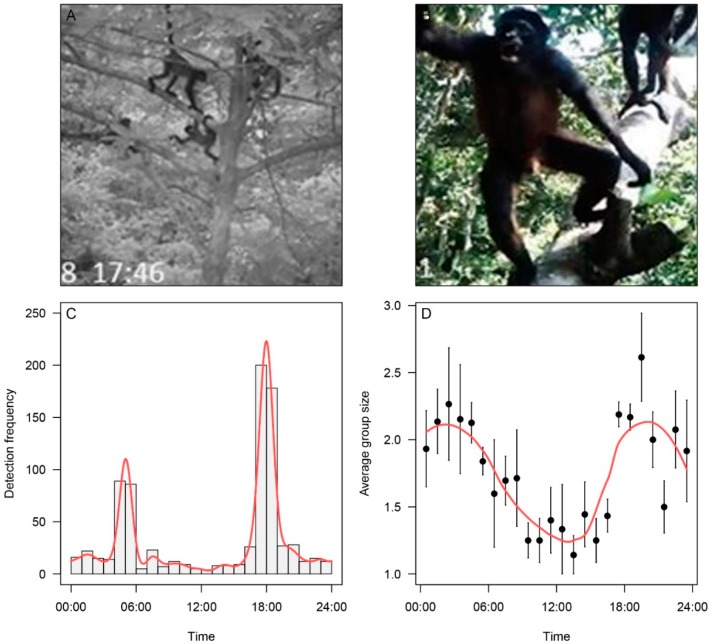
Arboreal camera trapping summary. (**A**) Spider monkey sub-group made up of females and juveniles congregating in sleeping site; (**B**) male spider monkey showing aggressive behavior; (**C**) diel raw camera trap detections of spider monkeys in sleeping sites; (**D**) diel pattern in average group size.

In total, we acquired data from 1055 trapping nights from the arboreal cameras, resulting in 830 observations of spider monkeys (e.g., Figure 2A,B), 521 of which were classified as independent. On average, sleeping sites were used by spider monkeys 8.4 days per month (min = 0.4, max = 22.5). Spider monkey activity at sleeping sites peaked between 04:00–06:00 and 17:00–19:00, with sporadic activity recorded both day and night (Figure 2C). The mean group size observed per capture event was 2.2 (min = 1; max = 7); group sizes were largest during the night, and smallest during the day (Figure 2D). Of the 1294 observations of individuals, 42% were identified as either male (*n* = 108) or female (*n* = 432). Adult female spider monkeys were identified by a conspicuous clitoris, while adult male spider monkeys could be identified by male genitalia and associated aggressive behavior. Adult spider monkeys that could not be assigned a sex were classified as unidentified (*n* = 754). Where juveniles were detected (*n* = 256), they were more likely to be observed with adult females (81% of detections events) than adult males (1% of detection events). We only assigned age classes between adult and juvenile spider monkeys, as deeper classification from dependent to infant and juvenile to sub-adult was deemed unreliable from the footage. Juvenile spider monkeys were classified as infants still clinging to adult females and semi-independent smaller-sized spider monkeys climbing in the presence of adult females but with no assistance. We did not assign a sex to juvenile spider monkeys (see [48] for further details and the Supplementary Videos for examples of adults, juveniles, and different sexes).

### 3.2. Terrestrial Vertebrate Activity Around Latrines

**Figure 3:**
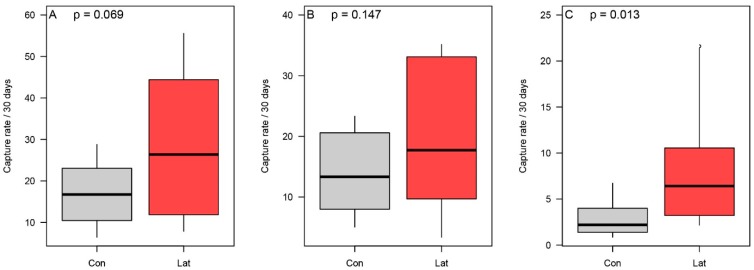
The comparison of overall captures at non-latrine controls (“Con”) and latrines (“Lat”) for all vertebrate detections (**A**), mammal detections (**B**), and bird detections (**C**).

We accrued 2287 trapping nights from terrestrial cameras (1154 nights within latrines and 1133 nights within non-latrine controls), which resulted in 4348 raw records of medium-to-large vertebrate species within latrines versus 2121 records in non-latrine controls. Nineteen species of mammal, nine species of bird, and a single species of reptile were detected at latrine cameras, and eighteen species of mammal and six bird species were detected at the non-latrine controls (see Supplementary Table S2 for a complete list). Looking at general visitation rate patterns, on average, latrines had higher vertebrate visitation rates (relative increase = +97%; Figure 3A; Supplementary Table S3), higher mammal visitation rates (+75.8%; Figure 3B; Supplementary Table S3), and higher bird visitation rates (+205.6%; Figure 3C; Supplementary Table S3); however, there was only statistical support for the differences in the case of birds (Figure 3; Supplementary Table S3; see Supplementary Table S4 for mean independent visitation rates for groups and species).

**Figure 4:**
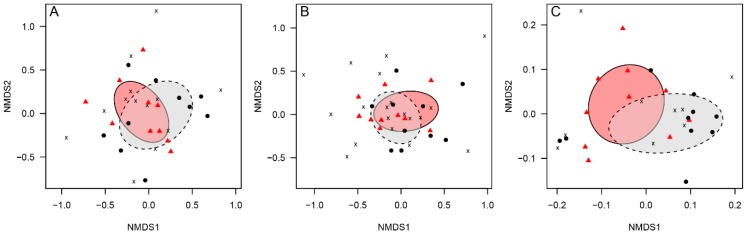
Non-metric multidimensional scaling (NMDS) plots of medium–large vertebrates (**A**), dung beetles (**B**), and soil parameters (**C**). Black circles = non-latrine controls; red triangles = latrines; *x* = species-specific loadings; grey polygon and dashed line = control community ellipse; red polygon and solid line = latrine community ellipse.

Although the vertebrate community compositions at latrine and non-latrine sites were similar (marked overlap in the community-specific ellipses; Figure 4A), the differences between the two communities were significant (F = 2.33, *p* = 0.03). The difference in composition was principally driven by three species: The paca (*Cuniculus paca*), which was found at a rate 427.2% higher in latrines than non-latrine controls (Χ^2^ = 6.79; *p* = 0.009; Supplementary Table S3); the great curassow (*Crax rubra*), found at a rate 212.2% higher than non-latrine controls (Χ^2^ = 4.75; *p* = 0.029; Supplementary Table S3); and the agouti (*Dasyprocta punctata*), which was found at a rate 68.6% higher than non-latrine controls, although this difference was not statistically significant (Χ^2^ = 1.238; *p* = 0.266; Supplementary Table S3).

### 3.3. Dung Beetle Activity Within Latrine Sites

**Figure 5:**
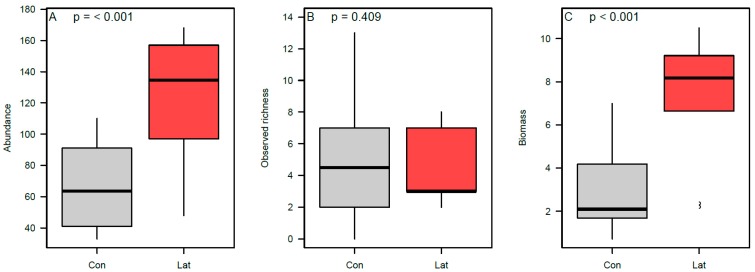
The differences in dung beetle species richness, abundance, and biomass at latrines and non-latrines; (**A**) dung beetle species richness per trap, (**B**) dung beetle abundance per trap, and (**C**) dung beetle biomass per trap (g); where “Con” = control sites and “Lat” = latrine sites.

We accrued a total of 1783 dung beetle captures of 22 species: 1192 captures representing all 22 species detected in the study were found in latrine sites and 591 captures representing 15 species were detected in non-latrine controls (see Supplementary Table S5 for a species list and respective mean dry biomass). On average, latrines had higher overall dung beetle captures than paired non-latrine controls (relative increase = +77%; Figure 5A; Supplementary Tables S3 and S6), higher observed richness (+35%; Figure 5B; Supplementary Tables S3 and S6), and greater total biomass (+374.8%; Figure 5C; Supplementary Tables S3 and S6). However, there was no statistical support for the difference in observed richness. There was also support for community-level differences in the dung beetle assemblages between latrine and control sites (Figure 4B; F = 3.57, *p* = 0.01), principally driven by three species. *Canthon aequinoctalis* was found at a rate 265.6% higher in latrines than non-latrine controls (Χ^2^ = 12.20; *p* = 0.0005; Supplementary Table S3); *Onthophagus batesi* was found at a rate 68.8% higher than in non-latrine controls, although this was not statistically significant (Χ^2^ = 1.50; *p* = 0.220; Supplementary Table S3); and *Onthophagus prascellens* was found at a rate 14.9% higher than non-latrine controls, although this difference was also not statistically significant (Χ^2^ = 0.12; *p* = 0.733; Supplementary Table S3).

### 3.4. Latrine Floristic Composition and Soil Parameters

**Figure 6:**
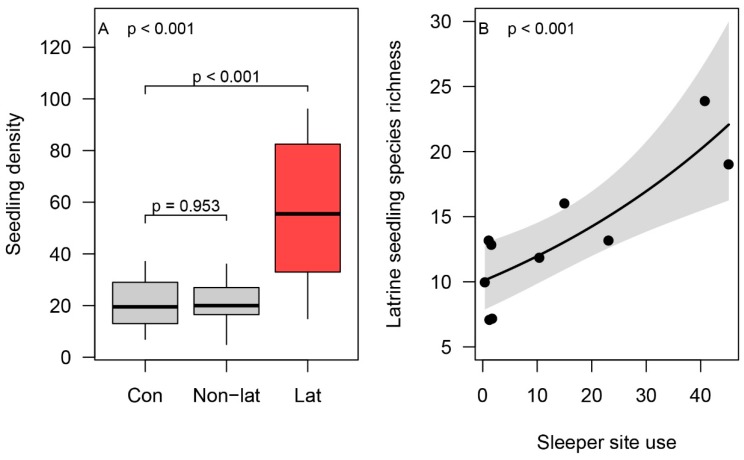
(**A**) Seedling density by quadrat location. Where “Con” = control quadrat outside of tree crown; “Lat” = latrine location within tree crown; “Non-lat” = non-latrine location within tree crown. (**B**) Plant species richness vs. sleeping site use: Where points = latrine observed seedling species richness; black line = mean relationship between use and seedling richness; and grey shading = 95% confidence interval.

Latrine sites had significantly higher seedling densities than control sites (Figure 6A). This effect was localized in the latrines (and not the total area beneath sleeping sites), as non-latrine sites beneath sleeping sites showed no detectable difference in seedling density than control sites (Figure 6B). A total of 80 different seedling species from 28 families were recorded in latrines. Furthermore, spider monkeys were directly observed consuming 50 species of plant from 27 different families during in-situ follows. Considering sapling inventories and direct observations together, the total diversity of plants dispersed by spider monkeys was 111 species (see Supplementary Tables S7 and S8). Only 19 species were detected in both latrines and via direct visual observations. The visitation rate of latrines by spider monkeys was positively correlated with seedling species richness within the latrine sites (Figure 6B).

Although there was marked visual separation of soil parameters between latrine sites and non-latrine controls (Figure 4C), this difference was not statistically significant (F = 0.904, *p* = 0.417). Consistent with this, of the two soil parameters suggested to drive the differences between latrines and non-latrine controls, only one showed a statistically significant change in concentration: Manganese was present at 22.7% lower concentration than in controls (Χ^2^ = 15.0; *p* = 0.0001; Supplementary Table S3); there was no statistically significant change in iron concentration (Χ^2^ = 0.87; *p* = 0.349; Supplementary Table S3). Of the three parameters thought to be key limiters on plant growth in the tropics [42]: Potassium was found in increased concentrations at latrine sites (Χ^2^ = 0.5.442; *p* = 0.020; Supplementary Table S3); there was no detectable difference in nitrogen levels (Χ^2^ = 0.0741; *p* = 0.785; Supplementary Table S3); and the assay was not sensitive enough to detect meaningful variation in phosphorus levels.

## 4. Discussion

We used remote camera traps both at ground-level and in the canopy, and used baited pitfall traps, to highlight the ecological impacts of spider monkey sleeping sites and their associated latrines. In summary: (1) Arboreal cameras allowed us to characterize spider monkey sleeping site use and composition; (2) terrestrial cameras allowed us to confirm that latrines attract greater vertebrate visitation rates, some of which are important secondary seed dispersing species; (3) baited pitfall traps indicate that latrines associated with sleeping sites attract a greater abundance and biomass of secondary seed-dispersing dung beetles that shape brown food webs; (4) floristic analysis of latrines and direct observations determined a total of 111 plant species consumed by spider monkeys (over half of these were detected by assessment of the 10 surveyed latrines; easier and less labor-intensive than direct observations (e.g., Scherbaum and Estrada [49] observed only 42 species of plants consumed in 6 months of field work)); and (5) chemical analysis of soils showed that latrines create heterogeneity in the potassium and manganese levels of rainforest soils. We believe this work highlights that the high fidelity of spider monkeys to sleeping sites leads to a hotspot of ecological interaction and activity which may have substantial downstream impacts on rainforest floristic composition and maintenance of diversity. We discuss each point in turn below.

As with previous research [8,11,50], we found that sleeping sites were typically large diameter trees, with horizontal thick limbs and that reach heights of up to and over 40 m. Studies to date on *Ateles* sleeping sites have been carried out in Santa Rosa National Park (Costa Rica), Manu National Park (Peru), and Calakmul, in the Lacandonian forest of Mexico [6,8,11,51,52,53]. The average sleeping site density at breast height (DBH) detected in Osa (59.5 cm) was 15.6 cm lower than that detected by Chapman (75.1 cm) [8] and just 2.9 cm less that the 62.4 cm determined in the continuous forest areas surveyed by González-Zamora et al. [11]. Although some trees were of the same genus as those identified in the Lacandona forests of Mexico (*Dialium* and *Brosimum*), we also detected many different species and genera being utilized; including fast-growing figs, *Tachigali* and *Symphonia*, and ancient old-growth species of *Caryocar, Hieronyma,* and *Vantanea*. Many of the species we identified spider monkeys sleeping in represent their own key food resources (see González-Zamora et al. [54]; and the list of species in Supplementary Table S7). In terms of saplings and seeds in latrines, there were some similar species of seeds identified in Howler monkey latrine sites from French Guiana [55]. As other large Neotropical primates also display sleeping site latrine behavior, it would be advantageous to investigate the latrines of both species at the same study location and season to make a direct comparison of food specialization to further our general understanding of latrine dynamics of Neotropical primate communities. Sleeping site activity has been documented in Neotropical night monkeys sleeping in family groups, often in cavities [56], but no latrines were detected or discussed. Sleeping site usage of gibbons and macaques in the old-world forests has been explored [57,58], but both groups showed weak evidence of sleeping site fidelity; a likely reason why latrines do not accumulate under such conditions and have not been investigated. The need to move regularly in response to predator pressure was a likely explanation for transiency in these systems. Lemurs do establish latrines, often located near to sleeping sites [59,60]. They are not however restricted to or directly below sleeping sites, and it is suggested that the latrines offer a form of communication to defend specific resources within the habitat (such as a sleeping or foraging site) and for information exchange [60]. This suggests that primates under different environmental and predation pressures display different social and sleeping behaviors. This results in diversified dispersal mechanisms that likely shape forest systems in different ways.

Activity patterns of spider monkeys have previously been restricted to assessments during diurnal hours and made via direct observations (for example, see Wallace [61]). By using arboreal camera traps, we were able to determine inferences in the 24-hour activity pattern within their sleeping sites. Spider monkeys spend up to four hours a day within their sleeping sites, with activity peaking in around dusk. This is likely an important time for socializing, such as interacting with group members in grooming activities that might be less feasible during foraging movements throughout the day [62]. Group sizes were generally greater on cameras in these sleeping-aggregating hours. One limitation of our study was the use of a single camera. While this is a common limitation in many camera trap studies generally, it might be advantageous to have multiple cameras to ensure that any monkeys using the sleeping site are recorded and do not get missed. Testing this using multiple cameras in sleeping sites could validate the use of a single camera versus the added value of multiple (i.e., how much extra data are gathered for additional financial cost). 9We also observed clear evidence of nocturnal activity for a species previously thought to be strictly diurnal [19,63,64]. This nocturnal activity could represent vigilance behavior [65], or potentially the nursing of young individuals [65]. A more complete picture of 24-hour activity might be gathered with camera traps also placed within foraging trees surrounding the sleeping sites to better represent daily activity patterns. The cameras detected the first video evidence (to the best of our knowledge) of a vampire bat attempting to climb onto (potentially to feed) on a spider monkey’s tail in their sleeping site (see Supplementary Videos).

In addition to understanding the activity within sleeping sites, arboreal camera traps also allowed us to investigate the social dynamics of the groups using the trees, and to determine that males generally slept in different sleeping sites to females and young offspring. This segregation agrees, to some extent, with Chapman [8], who suggests that males are less likely to use regular sleeping sites than females. Chapman suggests that females are generally more likely to return to regular sleeping sites, while larger males are more likely to spend a night close to their foraging resources. Although we did not investigate this here, arboreal camera traps have the potential to address this question by covering a greater number of sleeping sites, over a greater timeframe. The other challenge within this study was being able to identify sexes of the individuals in the video footage. Although a large proportion of individuals could not be determined, 540 individuals were identified as male or female, which is still a relatively high number. Once again, additional cameras might help to increase the number of individuals that can be accurately assigned a sex, as could the continuous improvement of cameras making it easier to clearly determine sexes from higher quality imagery.

This is the first time that spider monkey latrines have been directly proven to present a large and diverse food resource that is attractive to seed predators and secondary dispersers [13]. Although the latrines appear to attract a significant number of seed predators (e.g., small mammals), some plant species dispersed by monkeys have been shown to recruit well at latrines [11]. This is likely a result of the constant rain of seeds in the area, and as they also provide high-quality food patches for secondary dispersers such as curassows and dung beetles. Dung beetles provide a dual service in terms of dispersing and burying seeds, thus increasing the likelihood of successful germination [24,25,28,29,66] by reducing over clumping of seeds and saplings. In addition to seed predators and dispersers, some species attracted to the latrines were carnivores (e.g., ocelot) and omnivores (e.g., coatis), likely attracted to the latrines to prey upon the abundance of other species and invertebrates drawn to the area. This attraction of different guilds deserves further attention to fully characterize the downstream effects of spider monkeys and their respective latrines [55].

The potential for additional nutrient resources available as a result of feces deposition in latrines was also identified by Pouvelle et al. [55] with Howler monkeys. Our data suggest that although the overall soil property composition was not substantially different, latrines could still influence the uptake of specific tree growth-limiting nutrients, in particular potassium [42,67,68]. The higher pH levels detected in latrines (although non-significant) could be responsible for inhibiting levels of manganese release [69]. In addition to the value of potassium for plants, it is also a critical mineral resource for animal species visiting the latrines to forage. For example, white-lipped peccaries were observed on several occasions visiting the latrines and consuming seeds and saplings, and are known to need potassium (and other minerals), especially when females are lactating [70]. Spider monkey latrines therefore likely provide a rainforest-limited resource to a recognized ecosystem engineer species [70].

Future work across more sleeping sites and a greater number of latrines would be able to further corroborate generally the value of Ateline (and other primate) sleeping–latrine sites for other rainforest wildlife and plants. Identifying sleeping sites and latrines was particularly difficult, as it took a significant amount of field time (three months) to detect the sleeping sites. Following large primates off-trail and in low light conditions is challenging and poses some safety risks. An effective solution to this could be to utilize drones and thermal cameras to detect spider monkeys in their sleeping sites [71]. This could speed up latrine detection and provide precise counts of individuals to complement the footage from the in-situ cameras.

## 5. Conclusions

This work adds support to the influential role which spider monkeys play in the formation of tropical forest diversity. Tropical forests without spider monkeys (either as a result of local hunting pressures or extirpation following habitat clearance; Peres et al. [1]) will likely be more homogeneous than locations with spider monkeys, and will never contain the same composition of old-growth primary forests [2,72]. The insights provided from the camera traps were made even more powerful through the complementary use of traditional plant and pitfall trap censuses. Although large rainforest trees are likely important sleeping sites to spider monkeys for a variety of reasons, such as providing safe spaces to rest from predators [58,73], and as key sites for social interactions and relationship building between groups members [55,58], they also appear to play an important role in shaping forest community structure. This is aided by terrestrial secondary dispersers attracted to the high-quality resources provided by the latrines (saplings, insects, minerals, and fruits). The case for describing Ateline primates as rainforest ecosystem engineers [74,75] is increasingly compelling, as our evidence suggests that they modulate the availability of resources to other species by causing physical changes in both biotic and abiotic materials. Such species should be of principal conservation concern if we hope to maintain Neotropical forest integrity in a rapidly changing environment.

## Supplementary Materials

The following are available online at https://www.mdpi.com/2076-2615/9/12/1052/s1. Table S1: Sleeping trees recorded during the study period belonged to 21 different species, 18 genera and 12 families. Table S2: Species list for each group (mammals, birds and reptiles) recorded by the terrestrial cameras in latrines, non-latrines and by the arboreal camera traps in the sleeping sites. Table S3: Summaries and effects sizes for the linear mixed effects models on latrines and non-latrine controls. Where: ‘Lower 95% CI’ = lower 95% confidence interval; ‘Upper 95% CI’ = upper 95% confidence interval, ‘Effect size’ is estimated percentage change in the response variable from the control site, to the latrine. Emboldened rows represent confidence intervals that don’t overlap zero. Table S4: Mean independent visitations and standard error for terrestrial camera trap result for latrines and non-latrine controls for each group, or species where there were ≥10 independent detections. Table S5: Mean biomass (measured in grams) for each dung beetle species (calculated from previous dung beetle research at study site) which was used to calculate total biomass of each pitfall trap for this study. Table S6: Abundance, species richness and biomass of dung beetles for each of the sampled latrine and non-latrine control sites during the study period. The biomass was calculated from biomass measurements recorded in a previous study in 2017. An average biomass was calculated based on measurements for 1–25 individuals for each species this was then used to calculate an overall biomass for each trapping location. Table S7: Floristic diversity in spider monkey latrines shows they are a sapling and seed hotspot. A total of 111 species linked with spider monkeys feeding; 80 of the species were found from investigating latrines and 50 were identified from direct feeding observations (observations made from August 2018 to April 2019). Table S8: Species list of food consumed by spider monkeys observed in latrines and by direct visual feeding observations (all observations made from August 2018 to April 2019). To investigate the seedling richness in the latrines, a 1m2 quadrat was surveyed in the centre of each sub-selected latrine. In each quadrat, all the seedling species were counted and identified at the lowest taxonomic level possible, once a month for nine months (August 2018 to April 2019).
