# Supplementary material for: Spider Monkeys Rule the Roost: Ateline Sleeping Sites Influence Rainforest Heterogeneity
Source: Animals (Basel). 2019 Dec 1;9(12):1052. doi: 10.3390/ani9121052 (PMC6941026; doi:10.3390/ani9121052)
Supplement: Supplementary file 1 [file animals-09-01052-s001.pdf]

Supplementary Materials

Supplementary Video links

Spider monkeys: https://www.youtube.com/watch?v=vDIPW172aDs&feature=youtu.be

Secondary dispersers and seed predators: https://www.youtube.com/watch?v=tnhUsz39R7o

Table S1 - Sleeping trees recorded during the study period belonged to 21 different species, 18 genera and 12 families.

Sleeping site	Family	Tree species	DBH (cm)	Latrine identified	Ten Further Monitored
Sleeping site 1	Anacardiaceae	*Tapirira guianensis*	20.7	x	
Sleeping site 2	Malvaceae	*Luehea seemannii*	44.3		
Sleeping site 3	Clusiaceae	*Symphonia globulifera*	24.5		
Sleeping site 4	Moraceae	*Ficus insipida*	115	x	x
Sleeping site 6	Humiriaceae	*Vantanea barbourii*	65		
Sleeping site 8	Fabaceae	*Tachigali versicolor*	109.8	x	x
Sleeping site 9	Sapotaceae	*Pouteria sp.*	75.1	x	
Sleeping site 10	Clusiaceae	*Symphonia globulifera*	58	x	
Sleeping site 11	Myristicaceae	*Otoba novogranatensis*	27.5		
Sleeping site 12	Humiriaceae	*Vantanea barbourii*	85	x	
Sleeping site 13	Fabaceae	*Dialium guianense*	55	x	x
Sleeping site 14	Moraceae	*Pseudolmedia spuria*	24.2	x	
Sleeping site 15	Myristicaceae	*Otoba novogranatensis*	36.5		
Sleeping site 16	Myristicaceae	*Otoba novogranatensis*	41	x	x
Sleeping site 17	Anacardiaceae	*Tapirira guianensis*	47	x	
Sleeping site 18	Myristicaceae	*Otoba novogranatensis*	36.6		
Sleeping site 19	Burseraceae	*Tetragastris panamensis*	45.7		
Sleeping site 20	Moraceae	*Ficus osensis*	59.4	x	
Sleeping site 21	Moraceae	*Brosimum utile*	78	x	
Sleeping site 22	Fabaceae	*Dussia macroprophyllata*	80	x	
Sleeping site 23	Moraceae	*Brosimum utile*	89	x	
Sleeping site 24	Moraceae	*Brosimum utile*	89	x	
Sleeping site 25	Anacardiaceae	*Tapirira guianensis*	64.2		
Sleeping site 26	Fabaceae	*Tachigali versicolor*	72		
Sleeping site 27	Fabaceae	*Schizolobium parahyba*	160	x	
Sleeping site 28	Myristicaceae	*Virola spA*	40.1	x	x
Sleeping site 29	Clusiaceae	*Symphonia globulifera*	27.7	x	
Sleeping site 30	Myristicaceae	*Otoba novogranatensis*	66.4	x	x
Sleeping site 32	Moraceae	*Ficus insipida*	98	x	x
Sleeping site 33	Moraceae	*Ficus insipida*	35.2		
Sleeping site 34	Moraceae	*Ficus obtusifolia*	92	x	x
Sleeping site 35	Olacaceae	*Heisteria coccinea*	36.5	x	
Sleeping site 36	Humiriaceae	*Vantanea barbourii*	60	x	x
Sleeping site 37	Euphorbiaceae	*Hieronyma alchorneoides*	110	x	
Sleeping site 38	Moraceae	*Brosimum costaricanum*	40	x	
Sleeping site 40	Caryocaraceae	*Caryocar costaricense*	116	x	
Sleeping site 41	Anacardiaceae	*Tapirira guianensis*	78.9	x	
Sleeping site 42	Myristicaceae	*Otoba novogranatensis*	37.9	x	x
Sleeping site 43	Moraceae	*Ficus insipida*	62		

Table S2 - Species list for each group (mammals, birds and reptiles) recorded by the terrestrial cameras in latrines, non-latrines and by the arboreal camera traps in the sleeping sites.

**latrine**			**non-latrine**			**arboreal**		
**mammals**	**birds**	**reptiles**	**mammals**	**birds**	**reptiles**	**mammals**	**birds**	**reptiles**
Dasyprocta punctata	*Formicarius analis*	*Iguana iguana*	*Dasyprocta punctata*	*Formicarius analis*	*none*	*Ateles geoffroyi*	*Pteroglossus frantzii*	*Iguana iguana*
Dasypus novemcinctus	*Crax rubra*		*Nasua narica*	*Crax rubra*		*Cebus imitator*	*Crax rubra*	
Nasua narica	*Leptotila cassini*		*Dasypus novemcinctus*	*Geotrygon montana*		*Saimiri oerstedii*	*Pulsatrix perspicillata*	
Galictis vittata	*Leptotila plumbeiceps*		*Galictis vittata*	*Penelope purpurascens*		*Potos flavus*	*Ramphastos ambiguus*	
Panthera onca	*Geotrygon montana*		*Leopardus wiedii*	*Tinamus major*		*Bassaricyon gabbii*	*Glyphorynchus spirurus*	
Leopardus pardalis	*Garrulax leucolophus*		*Panthera onca*	*Aramides cajaneus*		*Alouatta palliata*	*Xiphorhynchus lachrymosus*	
Didelphis marsupialis	*Arremon aurantiirostris*		*Cebus imitator*			*Caluromys derbianus*		
Cebus imitator	*Tinamus Major*		*Didelphis marsupialis*			*Exilisciurus exilis*		
Philander opossum	*Catharus ustulatus*		*Philander opossum*					
Pecari tajacu			*Pecari tajacu*					
Tayassu pecari			*Tayassu pecari*					
Cuniculus paca			*Cuniculus paca*					
Puma conocolo			*Puma oncolor*					
Procyon cancrivorus			*Procyon cancrivorus*					
Conepatus semistriatus			*Tamandua mexicana*					
Tamandua mexicana			*Sciurus granatensis*					
Sciurus granatensis			*Tapirus bairdii*					
Sciurus variegatoides			*Eira barbara*					
Eira barbara								
**total species richness**			**total species richness**			**total species richness**		
**19**	**9**	**1**	**6**	**6**	**0**	**8**	**6**	**1**

Table S3 – Summaries and effects sizes for the linear mixed effects models on latrines and non-latrine controls. Where: 'Lower 95% CI' = lower 95% confidence interval; 'Upper 95% CI' = upper 95% confidence interval, 'Effect size' is estimated percentage change in the response variable from the control site, to the latrine. Emboldened rows represent confidence intervals that don't overlap zero.

**Method**	**Response**	**β-coefficient (latrine)**	**Lower 95% CI**	**Upper 95% CI**	**Effect size (%)**
Camera trapping	Total visitation rate	16.61	-1.53	34.74	+97.0
Camera trapping	Mammal visitation rate	10.62	-4.17	25.43	+75.8
**Camera trapping**	**Bird visitation rate**	**5.90**	**1.52**	**10.27**	**+205.6**
**Camera trapping**	**Paca visitation rate**	**1.96**	**0.56**	**3.36**	**+427.2**
**Camera trapping**	**Great curassow visitation rate**	**2.25**	**0.25**	**4.25**	**+212.2**
Camera trapping	Agouti visitation rate	7.31	-6.37	21.0	+68.6
**Beetle trapping**	**Dung beetle abundance**	**0.66**	**0.57**	**0.76**	**+76.5**
Beetle trapping	Observed richness	0.30	-0.02	0.62	+35.0
**Beetle trapping**	**Biomass**	**4.39**	**2.77**	**6.01**	**+374.8**
**Beetle trapping**	**Sp22 *Canthon aequinoctalis***	**7.35**	**3.30**	**11.40**	**+265.6**
Beetle trapping	Sp2 *Onthophagus batesi*	0.78	-0.47	2.04	+68.8
Beetle trapping	Sp7 *Onthophagus prascellens*	0.25	-1.19	1.69	+14.9
Soil parameters	Fe	-15.30	-48.5	17.9	-15.6
**Soil parameters**	**Mn**	**-0.26**	**-0.38**	**-0.12**	**-22.7**
**Soil parameters**	**K**	**0.38**	**0.07**	**0.71**	**+154.8**
Soil parameters	N	0.01	-0.07	0.06	-2.8

Table S4 – Mean independent visitations and standard error for terrestrial camera trap result for latrines and non-latrine controls for each group, or species where there were ≥10 independent detections.

Group/Species	Latrine or Non-latrine	Mean independent visitations	Standard error
All mammals	latrine	24.6	1.76
	non-latrine	14.01	0.89
All birds	latrine	8.8	0.62
	non-latrine	2.9	0.49
Tinamus major	latrine	2.6	0.22
	non-latrine	1.1	0.18
Crax rubra	latrine	3.3	0.25
	non-latrine	1.1	0.25
Dasyprocta punctata	latrine	17.9	1.64
	non-latrine	10.7	0.78
Nasua narica	latrine	0.7	0.05
	non-latrine	0.54	0.054
Dasypus novemcinctus	latrine	0.8	0.08
	non-latrine	0.23	0.043
Geotrygon montana	latrine	2.4	0.29
	non-latrine	0.42	0.22
Leopardus pardalis	latrine	0.2	0.03
	non-latrine	0.1	0.03
Didelphis marsupialis	latrine	0.16	0.34
	non-latrine	0.12	0.034
Tayassu pecari	latrine	0.42	0.05
	non-latrine	0.28	0.044
Pecari tajacu	latrine	0.18	0.04
	non-latrine	0.33	0.043
Tamandua mexicana	latrine	0.17	0.03
	non-latrine	0.13	0.03
Eira barbara	latrine	0.1	0.04
	non-latrine	0.34	0.036
small mammals and rodents	latrine	1.9	0.25
	non-latrine	0.5	0.04
Formicarius analis	latrine	0.18	0.03
	non-latrine	0.2	0.06
Cuniculus paca	latrine	2.4	0.19
	non-latrine	0.46	0.19

Table S5 – Mean biomass (measured in grams) for each dung beetle species (calculated from previous dung beetle research at study site) which was used to calculate total biomass of each pitfall trap for this study.

ID code	dung beetle species	mean biomass (g)
Sp1	*Onthophagus acuminatus*	0.006
Sp2	*Onthophagus batesi*	0.006
Sp3	*Onthophagus coriaceoumbrosus*	0.007
Sp7	*Onthophagus praescellens*	0.008
Sp11	*Dichotomius satanas*	0.216
Sp15	*Sulcophanaeus noctis*	0.285
Sp16	*Coprophanaeus pecki*	0.433
Sp17	*Coprophanaeus solisi*	0.233
Sp18	*Coprophanaeus telamon*	0.395
Sp19	*Deltochilum pseudoparile*	0.055
Sp20	*Deltochilum gibbosum*	0.505
Sp22	*Canthon aequinoctalis*	0.083
Sp23	*Canthon caelius*	0.005
Sp24	*Canthon humboldtii*	0.009
Sp29	*Scybalocanthon monilatus*	0.010
Sp30	*Canthidium aurifex*	0.009
Sp32	*Canthidium centrale*	0.013
Sp35	*Eurysternus cariibaeus*	0.035
Sp36	*Eurysternus foedus*	0.110
Sp37	*Eurysternus mexicanus*	0.006
Sp38	*Eurysternus plebejus*	0.006
Sp40	*Copris incertus*	0.057

Table S6 - Abundance, species richness and biomass of dung beetles for each of the sampled latrine and non-latrine control sites during the study period. The biomass was calculated from biomass measurements recorded in a previous study in 2017. An average biomass was calculated based on measurements for 1-25 individuals for each species this was then used to calculate an overall biomass for each trapping location.

site_id	abundance			spp richness			dry biomass (g)		
	latrine	control	total	latrine	control	total	latrine	control	TOTAL
**SS12**	104	28	132	10	6	10	5.767	0.159	5.926
**SS13**	143	99	242	7	7	8	6.775	4.475	11.250
**SS16**	88	28	116	12	5	12	5.792	0.231	6.022
**SS17**	157	61	218	12	9	12	7.664	2.726	10.390
**SS30**	137	28	165	10	8	11	9.419	0.669	10.088
**SS32**	39	66	105	6	4	7	0.483	0.710	1.193
**SS34**	151	88	239	8	6	9	6.490	2.132	8.622
**SS36**	121	55	176	10	6	10	6.714	0.495	7.209
**SS37**	98	35	133	11	8	12	7.134	1.799	8.933
**SS42**	105	32	137	7	6	8	7.272	1.753	9.025
**SS5**	93	36	129	8	4	8	2.326	0.129	2.455
**SS8**	160	98	258	9	11	12	8.047	3.778	11.825
**SS9**	133	66	199	9	8	11	4.307	2.842	7.149
**TOTAL**	**1529**	**720**	**2249**	**21**	**15**	**22**	**78.196**	**21.898**	**100.095**

Table S7- Floristic diversity in spider monkey latrines shows they are a sapling and seed hotspot. A total of 111 species linked with spider monkeys feeding; 80 of the species were found from investigating latrines and 50 were identified from direct feeding observations (observations made from August 2018 to April 2019).

Latrine ID	Sleeping Tree Family	Sleeping Tree Species	Number of food species identified
site 13	Fabaceae	*Dialium guianense*	14
site 16	Myristicaceae	*Otoba novogranatensis*	37
site 28	Myristicaceae	*Virola spA*	23
site 30	Myristicaceae	*Otoba novogranatensis*	46
site 32	Moraceae	*Ficus insipida*	12
site 34	Moraceae	*Ficus obtusifolia*	32
site 36	Humiriaceae	*Vantanea barbourii*	24
site 42	Myristicaceae	*Otoba novogranatensis*	27
site 5	Moraceae	*Ficus insipida*	15
site 8	Fabaceae	*Tachigali versicolor*	22
Total plants species identified in latrines			80
Species of plants consumed from direct observations			50
Total species of plants consumed			111

Table S8 - Species list of food consumed by spider monkeys observed in latrines and by direct visual feeding observations (all observations made from August 2018 to April 2019). To investigate the seedling richness in the latrines, a 1m^2 quadrat was surveyed in the centre of each sub-selected latrine. In each quadrat, all the seedling species were counted and identified at the lowest taxonomic level possible, once a month for nine months (August 2018 to April 2019).

Family	Species	Latrine	Direct observations
Anacardiaceae	*Mangifera indica*		x
Anacardiaceae	*Spondias mombin*	x	x
Anacardiaceae	*Spondias purpurea*		x
Anacardiaceae	*Tapirira guianensis*	x	x
Annonaceae	*Annonaceae 1*	x	
Annonaceae	*Duguetia confusa*	x	x
Annonaceae	*Guatteria amplifolia*		x
Annonaceae	*Guatteria pudica*	x	
Annonaceae	*Klarobelia stipitata*	x	
Annonaceae	*Oxandra venezuelana*	x	
Annonaceae	*Xylopia macrantha*	x	
Apocynaceae	*Lacmellea panamensis*	x	x
Araceae	*Philodendron sp.*		x
Arecaceae	*Attalea butyracea*	x	x
Arecaceae	*Bactris baileyana*	x	
Arecaceae	*Socratea exorrhiza*		x
Burseraceae	*Protium glabrum*		x
Burseraceae	*Protium sp1*	x	
Burseraceae	*Tetragastris panamensis*	x	x
Capparaceae	*Quadrella isthmensis*	x	
Celastraceae	*Cheiloclinium cognatum*	x	
Clusiaceae	*Clusiaceae 1*	x	
Clusiaceae	*Garcinia madruno*	x	
Clusiaceae	*Symphonia globulifera*		x
Dilleniaceae	*Dilleniaceae 1*	x	
Dilleniaceae	*Doliocarpus sp1*	x	
Elaeocarpaceae	*Sloanea picpica*		x
Fabaceae	*Bauhinia guianensis*	x	
Fabaceae	*Dialium guianense*	x	x
Fabaceae	*Dussia sp1*	x	
Fabaceae	*Fabaceae 1*	x	
Fabaceae	*Inga bella*	x	
Fabaceae	*Inga marginata*	x	
Fabaceae	*Inga multijuga*	x	x
Fabaceae	*Inga punctata*		x
Fabaceae	*Inga sapindoides*		x
Fabaceae	*Inga sp1*	x	
Fabaceae	*Inga sp2*	x	
Fabaceae	*Inga sp3*	x	
Fabaceae	*Inga sp4*	x	

Fabaceae	*Inga thibaudiana*	x	
Fabaceae	*Lonchocarpus sp.*		x
Humiriaceae	*Vantanea barbourii*	x	x
Lauraceae	*Beilschmiedia sp1*		x
Lauraceae	*Lauraceae 1*	x	
Lauraceae	*Nectandra umbrosa*		x
Lauraceae	*Ocotea sp1*	x	x
Malpighiaceae	*Byrsonima crispa*		x
Malvaceae	*Ochroma pyramidale*		x
Malvaceae	*Quararibea sp1*	x	
Malvaceae	*Theobroma angustifolium*	x	
Malvaceae	*Theobroma sp1*	x	
Marcgraviaceae	*Marcgravia schippii*		x
Melastomataceae	*Melastomataceae 1*	x	
Melastomataceae	*Miconia argentea*		x
Melastomataceae	*Mouriri gleasoniana*	x	
Meliaceae	*Guarea chiricana*	x	
Meliaceae	*Guarea pterorhachis*	x	
Meliaceae	*Guarea sp1*	x	
Meliaceae	*Trichilia septentrionalis*		x
Menispermaceae	*Hyperbaena leptobotryosa*	x	
Menispermaceae	*Menispermaceae 1*	x	
Moraceae	*Brosimum alicastrum*	x	
Moraceae	*Brosimum costaricanum*	x	
Moraceae	*Brosimum guianensis*	x	
Moraceae	*Brosimum lactescens*	x	x
Moraceae	*Brosimum utile*		x
Moraceae	*Castilla tunu*	x	
Moraceae	*Clarisia biflora*		x
Moraceae	*Ficus insipida*		x
Moraceae	*Ficus osensis*		x
Moraceae	*Ficus sp1*	x	x
Moraceae	*Ficus sp2*	x	
Moraceae	*Ficus sp3*	x	
Moraceae	*Moraceae 1*	x	
Moraceae	*Perebea hispidula*	x	
Moraceae	*Pseudolmedia spuria*	x	
Myristicaceae	*Compsoneura excelsa*	x	
Myristicaceae	*Compsponeura excelsa*		x
Myristicaceae	*Virola koschnyi*	x	x
Myristicaceae	*Virola macrocarpa*	x	
Myristicaceae	*Virola sebifera*	x	x
Myristicaceae	*Virola sp1*	x	
Myristicaceae	*Virola surinamensis*	x	
Myrtaceae	*Eugenia hammelii*		x
Olacaceae	*Heisteria acumuninata*	x	
Olacaceae	*Heisteria concinna*	x	x
Olacaceae	*Minquartia guianensis*	x	

Passifloraceae	*Erblichia odorata*		x
Phyllanthaceae	*Hieronyma alchorneoides*	x	
Rubiaceae	*Chione venosa*	x	x
Rubiaceae	*Genipa americana*	x	
Rubiaceae	*Rubiaceae 1*	x	
Rubiaceae	*Stenostomum turrialbanum*	x	
Salicaceae	*Laetia povedae*		x
Salicaceae	*Pleuranthodendron sp1*	x	
Sapindaceae	*Cupania rufescens*	x	
Sapindaceae	*Dilodendron costaricense*	x	
Sapindaceae	*Paullinia sp1*	x	
Sapotaceae	*Chrysophyllum cainito*		x
Sapotaceae	*Pouteria foveolata*	x	
Sapotaceae	*Pouteria sp1*		x
Sapotaceae	*Pouteria sp2*		x
Sapotaceae	*Pouteria torta*	x	x
Simaroubaceae	*Simarouba amara*	x	x
Smilacaceae	*Smilax sp1*	x	
Ulmaceae	*Ampelocera macrocarpa*		x
Urticaceae	*Cecropia insignis*		x
Urticaceae	*Cecropia sp1*	x	
Urticaceae	*Pourouma bicolor*	x	x
Vitaceae	*Cissus sp1*	x	
**Total species by type of observation**		**80**	**50**
